# The Divergent Effect of Different Infant Vaccination Schedules of the 13-Valent Pneumococcal Conjugate Vaccine on Serotype-Specific Immunological Memory

**DOI:** 10.3390/vaccines12091024

**Published:** 2024-09-07

**Authors:** Irene Tzovara, Ioanna Papadatou, Marianna Tzanoudaki, Christina Piperi, Christina Kanaka-Gantenbein, Vana Spoulou

**Affiliations:** 1 Immunobiology and Vaccinology Research Laboratory, School of Medicine, National and Kapodistrian University of Athens, 11527 Athens, Greece; iopapadatou@med.uoa.gr (I.P.); vspoulou@med.uoa.gr (V.S.); 2 First Department of Pediatrics, School of Medicine, “Aghia Sophia” Children’s Hospital, National and Kapodistrian University of Athens, 11527 Athens, Greece; ckanaka@med.uoa.gr; 3 Department of Immunology and Histocompatibility, Specialized Center and Referral Center for Primary Immunodeficiencies, “Aghia Sophia” Children’s Hospital, 11527 Athens, Greece; emainasgr@yahoo.gr; 4 Department of Biological Chemistry, School of Medicine, National and Kapodistrian University of Athens, 11527 Athens, Greece; cpiperi@med.uoa.gr

**Keywords:** *Streptococcus pneumoniae*, pneumococcal vaccination, pneumococcal conjugate vaccine, immunization schedule, immunological memory, memory B cells, antigen-specific memory, immunogenicity

## Abstract

Pneumococcal vaccination schedules are traditionally assessed based on the antibody response. The Memory B Cell (MBC) response has been less studied, despite its role in the magnitude and longevity of protection. We compared the immune response to different vaccination schedules with the 13-valent Pneumococcal Conjugate Vaccine (PCV13) and investigated the relationship between MBCs and the antibody response. Total and pneumococcal serotype (PS)-specific MBCs, their subsets and PS-specific IgG antibodies induced by a 3 + 0 (group A), 2 + 1 (group B) or 3 + 1 (group C) schedule in healthy infants were studied before and 1 month after the last PCV13. The relatively immature IgM+IgD+ MBC subset was the predominant subset in all groups but was larger in group A compared to group B and group C, indicating that age might be a significant parameter of the composition of the MBC pool. PS-specific MBCs at baseline were higher in group A, but they increased significantly only in the groups receiving the booster schedules (groups B and C). PS-specific IgM-only MBCs at baseline positively corelated with the antibody response and the PS-specific swIg MBCs post-immunization. Our findings illustrate the importance of a booster dose for the enrichment of PS-specific immunological memory. IgM-only MBCs and swIg MBCs may serve as additional correlates of vaccine-induced protection.

## 1. Introduction

*Streptococcus pneumoniae* remains a major cause of disease especially among young children, immunocompromised individuals, and the elderly, despite the significant decrease in invasive pneumococcal disease (IPD) observed after the implementation of universal infant immunization with Pneumococcal Conjugate Vaccines (PCVs) [1,2,3]. PCVs were initially licensed and introduced in 2000 in the USA for use in infants. They were administered with 3 primary doses followed by a booster dose (3 + 1 schedule), based on two large studies demonstrating high efficacy for this schedule [4,5]. Currently, a reduced schedule of PCV including 3 total doses is recommended by the World Health Organization (WHO) to protect children under 2 years of age. WHO supports the inclusion of PCVs in the routine immunization program of all countries, administered either as 3 primary doses without a booster (3 + 0 schedule) or as 2 primary doses with a booster dose (2 + 1 schedule) [6].

The reduction of four total doses to three is supported by (a) non-inferiority of antibody levels seen in the 2 + 1 versus the 3 + 1 schedule before and after the booster dose and (b) clinical trials demonstrating high antibody levels in the first year of life with an accelerated 3 + 0 infant schedule in developing countries, where disease rates are very high during this period [7,8,9,10]. In an effort to promote compliance and improve cost-effectiveness of national immunization policies, a trend to further decrease the number of primary doses is being considered in settings with high vaccine coverage and levels of herd immunity and has already been implemented in the UK, based on non-inferiority immunogenicity data following the booster dose [11,12,13,14,15].

Nevertheless, since the duration of protection is an important component of vaccine-induced protection, the ability of each schedule to establish immunological memory should also be considered for the longevity of immunity. Memory B cells (MBCs) play an important role in protection against encapsulated bacteria. They are responsible both for the rapid production of antibody-secreting plasma cells (PCs) upon antigen re-encounter, and the production of long-lived PCs that reside in the bone marrow, preserving long-lasting reservoirs of antibody-secreting cells [16]. Moreover, MBCs have the ability to maintain their own population, enhancing the longevity of protection [17,18].

More recently it has been shown that different phenotypes of MBCs fulfill distinct immunological destinies. Upon secondary challenge, IgM+ MBCs preferentially re-enter germinal center (GC) reactions to replenish the antigen-specific MBC pool by generating new IgM MBCs and switched Ig (swIg) MBCs with increased affinity. Pre-existing swIg MBCs act as effector cells rapidly differentiating to antibody-secreting PCs [19,20,21,22,23]. Lately, a subset of IgM+ MBCs that also expresses surface IgD (IgM + IgD + MBCs) has been gaining research interest and is considered a heterogeneous population comprised at least by MBCs formed outside the GC and marginal zone-like MBCs with an adaptive function that were formed early in the GC reaction [24,25,26]. This population is thought to be of lower affinity and have a shorter lifetime compared to the classical GC-experienced MBCs [27] but is generally not well characterized in humans and especially in young children.

In this study, we enumerate the Pneumococcal Serotype (PS)-specific MBCs, their subsets and the PS-specific IgG serum antibodies induced by a 3 + 0, a 2 + 1 or a 3 + 1 PCV13 immunization schedule in healthy infants in Athens, Greece. The aim of this study is to compare the effect of each schedule on the establishment of immunological memory, investigate the relationship between the different memory B cell subsets and the magnitude of the humoral response, and provide insights that could help determine the optimal immunization schedule for the establishment of long-term protection.

## 2. Materials and Methods

### 2.1. Study Participants and Study Design

**Figure 1:**
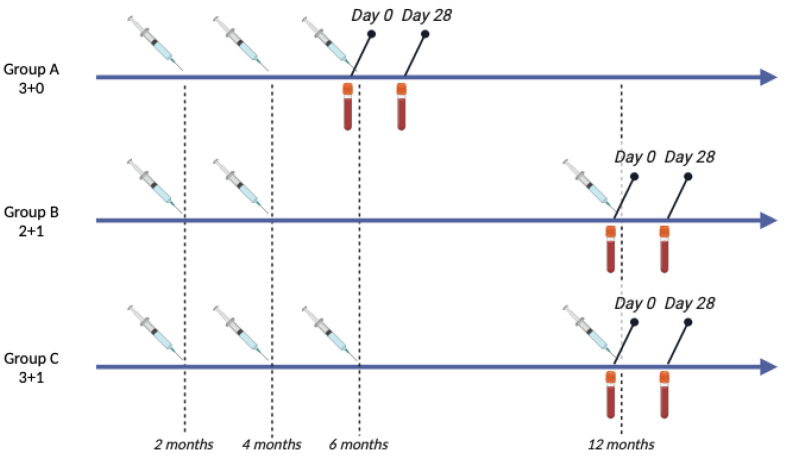
Study design. Infants were allocated to receive either 3 primary doses of PCV13 without a booster dose (group A, 3 + 0), 2 primary doses followed by a booster (group B, 2 + 1) or 3 primary doses followed by a booster (group C, 3 + 1). Serum and PBMC were collected before (day 0) and 28 days after (day 28) the last PCV13 dose of each schedule.

Forty-two healthy children aged 5–16 months of age were included in this study and were stratified into 3 groups. Group A (*n* = 17, 3 + 0 schedule) received 3 primary doses of PCV13 in the first year of life without a booster dose. Group B (*n* = 9, 2 + 1 schedule) received 2 primary doses of PCV13 in the first year of life followed by a booster dose 6 months later. Group C (*n* = 16, 3 + 1 schedule) received 3 primary doses of PCV13 in the first year of life followed by a booster dose 6 months later. The smaller sample size of group B was due to inadequate volume or quality of a number of samples collected at either timepoint which led to the complete exclusion of those children from the study. Children were also excluded from the study if they had received intravenous immunoglobulin (IVIG) in the past 6 months, experienced a severe allergic reaction to a previous vaccination, had a primary or secondary immunodeficiency, or had any other chronic condition. The study was approved by the hospital’s ethical committee and informed consent was obtained from the children’s parents prior to enrollment. Children received 3 or 4 doses of PCV13 according to their schedule, and blood samples were obtained before (day 0, D0) and 1 month (day 28, D28) after the last dose for each vaccination schedule (Figure 1).

### 2.2. PBMC and Serum Isolation

Serum samples were collected from the children and stored at −20°C. Peripheral Blood Mononuclear Cells (PBMCs) were isolated from whole blood that was drawn in EDTA tubes using blood-separation filter tubes (Kisker Biotech GmbH & Co., KG, Steinfurt, Germany) and cryopreserved in 10% DMSO (MilliporeSigma, Burlington, MA, USA) and 90% FBS (MilliporeSigma, Burlington, MA, USA). PMBC samples were frozen overnight at −80 °C in a freezing container (Nalgene^®^ Mr. Frosty^®^ Cryo 1 °C Freezing Container, Thermo Fisher Scientific, Waltham, MA, USA) and stored at −80°C, for a maximum of 12 months, until the batch was analyzed. RPMI 1640 medium (Gibco, Thermo Fisher Scientific, Waltham, MA USA), pre-warmed to 37 °C, was used to thaw the cryopreserved PMBCs, which were then washed twice (via centrifugation at 300 rcf for 5 min) with RPMI to improve cell recovery [28]. The thawed PBMCs were allowed to rest for 12–16 h at 37 °C in 5% CO_2_ in a freshly prepared cell resting buffer containing 90% IMDM (Gibco, Thermo Fisher Scientific, Waltham, MA, USA) and 10% FBS.

### 2.3. ELISA

Serum IgG serotype-specific antibodies were measured against pneumococcal serotypes (PS) 1, 3, 9 V and 19 A according to the WHO protocol of Pneumococcal Polysaccharide ELISA with a 007sp reference serum [29].

### 2.4. Flow Cytometry

Flow cytometry was used to identify the frequency and subset distribution of total and PS-specific memory B cells. For the detection of PS-specific B cells, a novel method was developed by our team, as described in a previous study [30]. Briefly, thawed PBMCs were stained using the PS1- and PS9V-multimer developed in-house and the following combination of monoclonal antibody fluorochromes: CD19-PC7, CD20-ECD, CD10-APC-AlexaFluor 750, CD27-PC5.5, anti-human IgM-PB and anti-human IgD-APC (clones J3-119, B9E9, ALB1, 1A4CD27, SA-DA4 and IA6-2 respectively, Beckman Coulter, Brea, CA, USA). Human FcR blocking reagent (Miltenyi Biotech, Bergisch Gladbach, Germany) was used to eliminate Fc-related non-specific binding of fluorochromes and PS-multimers. A 10 color/3 laser Navios EX flow cytometer (Beckman Coulter, Brea, CA, USA) was used, and FCS files were analyzed using Kaluza 2.1.1 software (Beckman Coulter, Brea, CA, USA).

Memory B cells (MBCs) were defined as CD19 + CD20 + CD10-CD27+ lymphocytes. The addition of CD20 further defined the B cell population (CD19+ lymphocytes), as it is not expressed on stem cells, pro-B cells and the vast majority of plasma cells [31,32]. Dual selection of CD20+ and CD10− B cells allowed the selection of mature B cells (CD10−) [33] while also minimizing non-specific signals, which is crucial when aiming to detect rare cell populations such as antigen-specific B cells. MBC subsets were defined as follows: “classical” switched Ig (swIg) MBCs as IgM-IgD- MBCs, IgM-only MBCs as IgM+ IgD- MBCs and “non-classical” MBCs as IgM+IgD+ MBCs. PS-specific B cells were detected within mature B cells according to the gating strategy previously described [30]. Gates for each MBC subset were defined for the total mature B cell population (Supplementary Figure S1) and the same gates were used to define the PS-specific B cell subsets.

### 2.5. Statistical Analysis

Variables were tested for normality using the Shapiro–Wilk test. IgG antibody levels were log-transformed prior to analysis to obtain normality and expressed as Geometric Mean Concentrations (GMCs, μg/mL) with 95% confidence intervals (95% CI). Memory B cell numbers and percentages were expressed as medians with interquartile ranges (IQRs). Antibody fold changes were calculated as the post/pre ratio and presented as medians with IQRs. Parametric tests were used to compare GMCs between the two timepoints and between groups and non-parametric tests were used for MBCs. Spearman’s rank correlation coefficient was calculated to relate IgG antibody levels with the percentage of PS-specific MBC subsets, and the different MBC subsets with one another. Statistical significance was set at 0.05 and all *p*-values reported are two-tailed. All the statistical tests and graphs were performed and created using GraphPad Prism software version 10 (GraphPad Software, San Diego, CA, USA).

## 3. Results

From the 42 children (25 boys, 59%) recruited in this study, 38 (90.5%) completed the follow-up, 1 month after receiving PCV-13. No demographic differences were observed among the three groups in terms of sex, race, number of siblings and daycare attendance. Samples from all children included in the study were available for measurement of their antibody response and samples from 32 (76%) children were available for the PS-specific MBC response study due to inadequate sample volume upon collection or poor sample quality upon thawing. Specifically for the PS-specific MBC assays, samples from both timepoints were available for 11 children from group A (3 + 0 schedule), 9 children from group B (2 + 1 schedule) and 12 children from group C (3 + 1 schedule).

### 3.1. Total Memory B Cell Population

**Figure 2:**
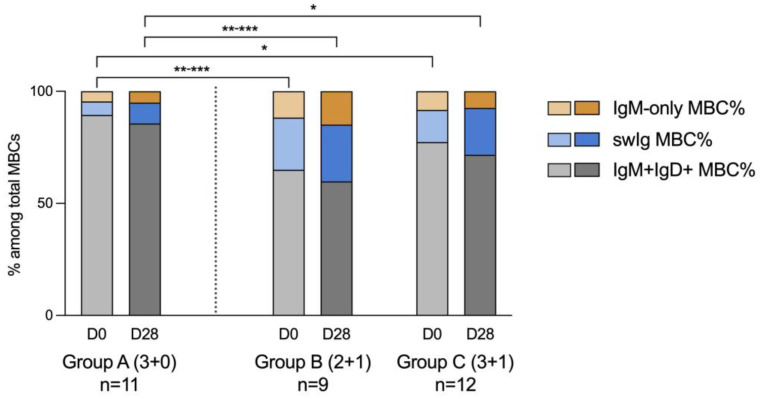
Distribution of total memory B cell (MBC) subsets. Mean percentage of IgM-only MBCs, swIg MBCs and IgM+IgD+ MBCs among total MBCs prior to the last PCV13 dose for each group (day 0, light-colored bars) and after 28 days (day 28, dark-colored bars). Comparisons between the 3 groups for each timepoint were performed using the Kruskal–Wallis test. Comparisons between the two timepoints for each group were performed using the Wilcoxon test. Statistical significance: * *p* ≤ 0.05, ** *p* ≤ 0.01, *** *p* ≤ 0.001.

The distribution of the MBC subsets differed significantly between subjects with or without a booster (Figure 2). Non-classical (IgM + IgD+) MBCs represented the main MBC subset in all study groups; however, at both timepoints this population was larger in group A when compared to groups Β and C (group A vs. B: *p* < 0.001; group A vs. C: *p* = 0.054 at D0, *p* = 0.067 at D28). Accordingly, infants in group A had significantly lower percentages of swIg MBCs compared to the other two groups, both before and 28 days after PCV13 (group A vs. B: *p* < 0.001, group A vs. C: *p* < 0.05). The percentage of IgM-only MBCs also differed between the groups and was the highest in group B at all timepoints compared to the two other groups (group B vs. A: *p* ≤ 0.009; group B vs. C: *p* = 0.06).

### 3.2. PS-Specific Memory B Cell Response

**Figure 3:**
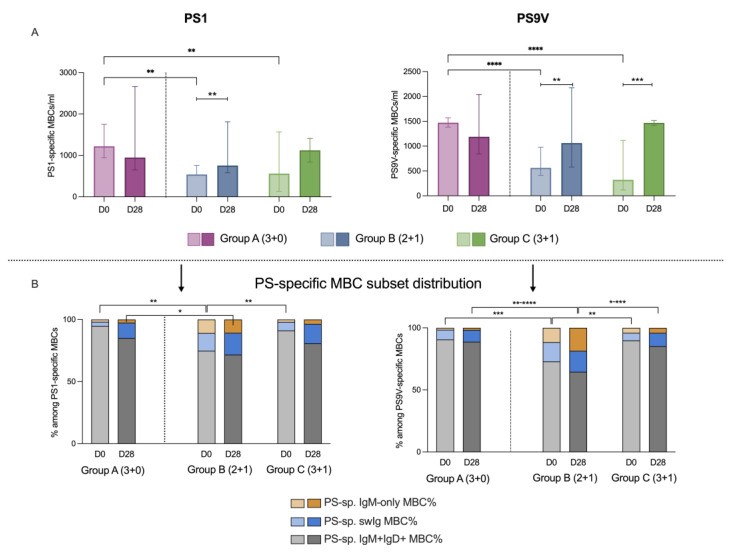
Kinetics and subset distribution of PS-specific MBCs. Absolute numbers (**A**) and subset distribution (**B**) of serotype-specific MBCs (cells/mL) in peripheral blood prior to the last PCV13 dose for each group (D0) and on day 28 post-immunization (D28) for serotypes 1 (left) and 9 V (right). The bars represent the median and the brackets represent the IQR. Comparisons between groups for each timepoint were performed using the Kruskal–Wallis test. Comparisons between the two timepoints for each group were performed using the Wilcoxon test. Statistical significance: * *p* ≤ 0.05, ** *p* ≤ 0.01, *** *p* ≤ 0.001, **** *p* ≤ 0.0001.

The number of PS-specific MBCs in the circulation (PS-specific MBCs/mL) on day 0 was significantly higher in group A compared to the other two groups for both PS1 and PS9V (Figure 3A). No significant differences in the numbers of PS-specific MBCs were observed between the groups on day 28. One-month post-PCV13 PS-specific MBCs increased in the schedules that included a booster (B and C) but decreased in group A. 

Similar to the total MBC subset distribution, the non-classical (IgM+IgD+) MBCs were the predominant phenotype of PS-specific MBCs for both serotypes tested at all timepoints and across all three schedule groups (Figure 3B). However, the percentage of PS-specific non-classical MBCs on day 0 was significantly lower in group B (76.36% of PS1-specific MBCs, 71.43% of PS9V-specific MBCs) compared to group A (94.64% of PS-1-specific MBCs, *p* < 0.001 and 90.2% of PS9V-specific MBCs, *p* < 0.001) and to group C (89.74% of PS-1-specific MBCs, *p* < 0.01 and 88.45% of PS9V-specific MBCs, *p* < 0.001). On day 28, the percentage of PS-specific non-classical MBCs against only serotype 9V was significantly lower in group B (65.33% of PS-9V-specfic MBCs) compared to group A (84.94% of PS-9V-specific MBCs, *p* < 0.01) and group C (84.21% of PS-9V-specific MBCs, *p* < 0.05). Accordingly, the IgM-only population was larger in group B for both serotypes and at both timepoints compared to the other two groups. Finally, the PS-specific swIg MBC population was also larger in group B compared to the other groups at both timepoints for both serotypes, though reaching statistical significance only at D0.

### 3.3. Antibody Response

**Figure 4:**
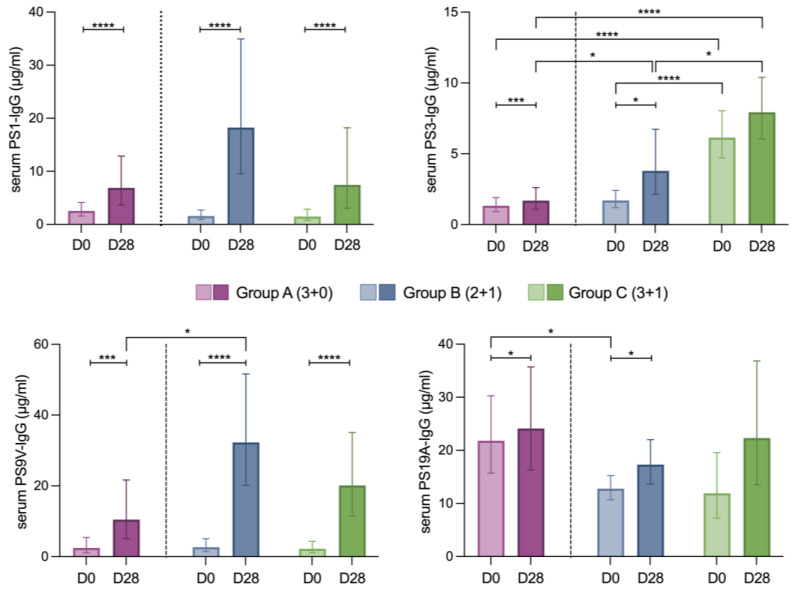
Antibody response to different PCV13 schedules. Serotype-specific IgG antibody concentrations (μg/mL) in serum prior to the last PCV13 dose for each group and on day 28 post-immunization for serotypes 1, 3, 9 V and 19 A). The bars represent the Geometric Mean Concentrations (GMCs) and the brackets represent the 95% Confidence Interval (95% CI). Ordinary one-way ANOVA was performed on log-transformed data (to obtain normality). Statistical significance: * *p* ≤ 0.05, *** *p* ≤ 0.001, **** *p* ≤ 0.0001.

All immunization schedules resulted in anti-polysaccharide IgG concentrations above the accepted protective threshold of 0.35 μg/mL [34] for all PS tested (Figure 4). Antibody concentrations increased significantly at D28 for the four tested serotypes across all vaccination groups. The number of doses in the primary schedule and the booster did not affect antibody levels after completion of each vaccination schedule, with the exception of PS 3 where children in group C had significantly higher antibody levels before and after the booster.

In contrast we were able to detect differences between different groups and serotypes with regards to the fold increase in antibody response, which is associated with pre-existing B cell memory (Table 1). Children who received PCV13 as the third dose of their primary series (group A) had a smaller fold change in serum antibody concentrations for all tested serotypes [fold change (IQR): PS1 1.5 (4.5), PS3 0.42 (0.6), PS9V 3 (5.5), PS19A 0.28 (0.4)] compared to children who received PCV13 as a booster dose [fold change (IQR), group B: PS1 7 (32), PS3 1.1 (2.2), PS9V 10 (14), PS19A 0.34 (0.45) and group C: PS1 4 (6), PS3 1.1 (2.2), PS9V 8 (7), PS19A 0.39 (0.82)]. This difference reached statistical significance only for serotypes 1 (*p* = 0.011) and serotype 9 V (*p* = 0.021), where fold increases were generally higher compared to PS3 and 19 A.

### 3.4. Correlation between Memory B Cell Response and IgG Serum Antibodies 

A significant correlation was observed between PS-specific IgM-only MBC% at D0 and the antibody response at D28 (for PS9V r = 0.65, *p* < 0.005 and for PS1 r = 0.46, *p* = 0.03). A similar correlation was observed between the PS-specific swIg MBC% at D0 and the antibody response at D28 for serotype 1 (r = 0.46, *p* = 0.03) and serotype 9 V (r = 0.42, *p* = 0.06). Finally, IgM-only PS-specific MBC% on Day 0 had a significant strong positive correlation with swIg PS-specific MBC% on Day 28 in the booster-groups (r = 0.76, *p* = 0.015 for PS1 and r = 0.8, *p* = 0.008 for PS9V). Interestingly, this correlation was not observed in group A.

## 4. Discussion

This is the first study, to our knowledge, to directly compare the immunological memory induced by the three commonly used PCV infant immunization schedules. In this study, we examined the phenotype composition of the memory B cell pool at each timepoint and enumerated the antigen-specific MBCs against vaccine serotypes induced by each of the three schedules.

In our study, the composition of the total MBC population differed significantly among the study groups and this difference could be age-related. While non-classical IgM+IgD+ MBCs constituted the main MBC subset in all infants, this population was significantly larger in the young infants of group A (6–7 months old) compared to the older children who received the booster schedules (≥12 months old). Thus, this relatively immature subset seems to reduce with age and be replaced by the more mature IgM-only and swIg MBCs in older children. Other study groups have also shown that the predominant MBC population in infancy is the IgM+IgD+ phenotype [25,35]. Blanco et al. have reported similar percentages of IgM+IgD+ MBCs in infants of 1–5 months of age that then gradually decline until 2 years of age [35]. Accordingly, germinal center formation is impaired early in infancy and improves gradually during the first 2 years of life [25,36].

The immunization schedule significantly affected the development of PS-specific memory. At baseline (D0), the number of circulating PS-specific MBCs was higher in group A compared to the groups including a booster at 12 months. This is probably due to the shorter interval from the previous dose in the 3 + 0 schedule (i.e., 2 months) compared to the booster groups (i.e., 8 months for the 2 + 1 schedule and 6 months for the 3 + 1 schedule), which seems to maintain a higher level of circulating memory B cells as opposed to the waning observed when several months have passed. Most importantly, PS-specific MBCs increased significantly at D28 only in children who received the booster dose, whereas when the third dose was given as part of the primary series at 6 months of age (group A), there was no significant change in the number of PS-specific MBCs post immunization, suggesting that a short interval between doses has a limited effect in the enrichment of PS-specific B cell memory [22,37]. Data from a study in Vietnam also suggests that a longer interval between PCV doses achieves better immunogenicity and prolongs protection after the first year of life [38]. Further investigations studying the antigen-specific memory response after different intervals between PCV doses are needed to confirm this relationship.

To better understand the effect of PCV13 on the formation of long-term immunological memory, we also examined the phenotypes of the PS-specific MBCs. In accordance with the phenotype distribution of total MBCs, PS-specific MBCs were predominantly of the IgM+IgD+ phenotype in all groups and timepoints but were higher in group A compared to groups B and C. This difference in the subset composition may explain the fact that PS-specific MBC numbers increased significantly only in the booster groups, as “non-classical” MBCs have limited ability to enhance and replenish the MBC pool due to their impaired engagement in GC reactions. Furthermore, high levels of circulating antibodies may also negatively influence the classical secondary memory response either by masking the antigenic epitopes or blocking the GC responses via a negative feedback mechanism [39,40]. Overall, there seems to be a benefit with longer intervals between serial vaccinations as per schedules including a booster dose. Our findings are supported by epidemiological evidence from Africa, where 3 + 0 schedules are not effective against IPD after the first year of life, especially for challenging serotypes such as serotype 1 [41]. However, the induction, maintenance and reactivation of memory B cells is regulated by many factors including antigen exposure, T cell interactions and cytokine signaling. Thus, further research should aim to incorporate the simultaneous assessment of these factors e.g., with a systems biology approach.

The number of doses in the primary series did not influence significantly the antibody levels following the booster dose for all tested serotypes with the exception of PS3. For this challenging serotype, children in the 3 + 1 group reached significantly higher antibody levels compared to the 2 + 1 group. This finding is in accordance with epidemiological evidence that PS3-related breakthroughs and vaccine failure cases occur more often in the 2 + 1 schedules [42].

In contrast, we observed some interesting differences when the antibody response was examined in terms of the fold increase of the serum antibody concentrations. Larger antibody fold increases were observed when the last dose was administered at an older age, i.e., we were able to detect differences in the antibody fold increase for all tested serotypes when the last dose was administered at an older age and, for serotypes 1 and 9V, between different immunization schedules. Such differences, which could not be captured by the antibody snapshot taken at specific time following vaccination, could potentially conceal immunological gaps induced by different immunization schedules with potential biological significance [41].

Finally, we investigated whether MBCs could serve as an alternative correlate of protection capable of predicting the magnitude of vaccine-induced long-term protection by studying the relationship between the PS-specific MBC subsets and the antibody response. A positive correlation was observed between the classical swIg at baseline and the antibody response, which is in accordance with their role as effector cells, rapidly differentiating into antibody secreting cells upon secondary challenge [20,22]. Moreover, IgM-only PS-specific MBCs at baseline positively correlated both with the antibody response and the swIg PS-specific MBCs at 1 month. Notably, this correlation was observed even though their populations both increased between the two timepoints, supporting a role of IgM MBCs in expanding the swIg MBC population while also preserving the IgM MBC pool by re-entering the GC reactions [19,22,23].

This study is subject to three main limitations. The first limitation is the relatively small sample size. A larger sample size may have revealed more significant differences between the groups, especially regarding the MBC response for which the sample size was smaller due to technical limitations. Moreover, the small number of studied serotypes did not allow us to address possible immunity gaps against specific serotypes with distinct immunological properties like serotype 3. Including multiple serotypes would ensure a more thorough evaluation of the vaccine-induced memory response against pneumococcal disease which is essential in tailoring informed vaccine strategies that ensure broad protection. Finally, the MBC subsets were phenotypically defined using a limited number of cell surface markers. As advances in the field of immunobiology have been accelerating in recent years and reagents are becoming more available and affordable, the novel flow cytometry method used in this study could be adapted to mass cytometry which allows for a significantly larger number of markers to be included.

## 5. Conclusions

Altogether, this study illustrates the importance of a booster dose in the formation of a robust PS-specific immunological memory. The optimal vaccination schedule for long-lived protection against pneumococcus should consider not only the number and interval of doses but also their timing, as age may be a significant parameter for the MBC pool composition. The study of PS-specific Memory B cells, and particularly IgM-only and swIg MBCs, together with antibodies will allow for a more in-depth quantitative and qualitative evaluation of different vaccination schedules and future vaccine formulations.

## Figures and Tables

**Table 1:** Serotype-specific IgG concentrations with fold changes between day 0 and day 28.

	Day 0 GMCs (95% CI)	Day 28 GMCs (95% CI)	Fold Change Median, IQR	*p*-Value *
Serotype 1				
Group A	2.6 (1.6–4.1)	6.9 (3.7–12.9)	1.5 (4.5)	**0.011**
Group B	1.6 (0.9–2.7)	18.3 (9.5–34.9)	7 (32)	
Group C	1.5 (0.8–2.9)	7.5 (3.1–18.2)	4 (6)	
Serotype 3				
Group A	1.3 (0.9–1.9)	1.7 (1.1–2.6)	0.42 (0.6)	0.11
Group B	1.7 (1.2–2.4)	3.8 (2.1–6.7)	1.1 (2.2)	
Group C	6.2 (4.7–8)	7.9 (6–10.4)	0.39 (0.56)	
Serotype 9 V				
Group A	2.5 (1.2–5.4)	10.5 (5.1–21.7)	3 (5.5)	**0.021**
Group B	2.7 (1.4–5.1)	32.3 (20.2–51.6)	10 (14)	
Group C	2.2 (1.1–4.3)	20.1 (11.6–35.1)	8 (7)	
Serotype 19 A				
Group A	21.8 (15.7–30.3)	24.1 (16.3–35.7)	0.28 (0.4)	0.29
Group B	12.8 (10.7–15.2)	17.3 (13.6–22)	0.34 (0.45)	
Group C	11.9 (7.2–19.6)	22.3 (13.5–36.9)	0.39 (0.82)	

* Comparisons of fold change among groups, performed using the Kruskal–Wallis test; Group A = 3 + 0 PCV13 schedule, Group B = 2 + 1 PCV13 schedule, Group C = 3 + 1 PCV13 schedule.

## Data Availability

Further information about data supporting the reported results will be provided by the corresponding author upon reasonable request.

## Supplementary Materials

The following supporting materials can be downloaded at: https://www.mdpi.com/article/10.3390/vaccines12091024/s1, Figure S1: Gating strategy of memory B cell (MBC) subsets in thawed Peripheral Blood Mononuclear Cells (PBMCs) from young children vaccinated with 3 or 4 total doses of PCV13.
