# Supplementary material for: The Divergent Effect of Different Infant Vaccination Schedules of the 13-Valent Pneumococcal Conjugate Vaccine on Serotype-Specific Immunological Memory
Source: Vaccines (Basel). 2024 Sep 7;12(9):1024. doi: 10.3390/vaccines12091024 (PMC11435716; doi:10.3390/vaccines12091024)
Supplement: Supplementary file 1 [file vaccines-12-01024-s001.zip › vaccines-3169318-supplementary.pdf]

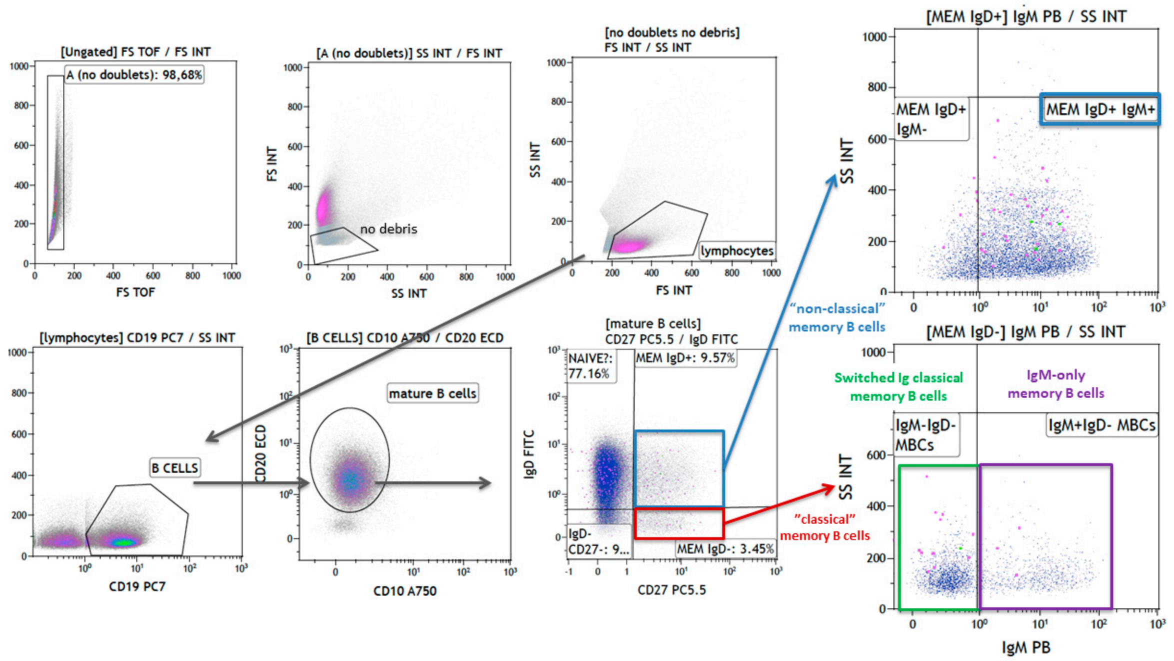

***Supplementary figure S1. Gating strategy of memory B cell (MBC) subsets in thawed Peripheral Blood Mononuclear Cells (PBMCs) from young children vaccinated with 3 or 4 total doses of PCV13**. A forward scatter height (FSC-H) versus forward scatter area (FSC-A) density plot was used to exclude doublets. An FSC versus SSC density plot was used to exclude debris and identify lymphocytes. B cells were initially identified as CD19+SSC signals on gated lymphocytes and mature B cells were characterized as CD20+CD10-B cells. Using a CD27 vs. IgD density plot on mature B cells we identified CD27+ cells as MBCs and characterized them as either "classical" (IgD-) or "non-classical" (IgD+). We further divided "classical" MBCs with an IgM vs. SSC plot into switched Ig (swIg) classical MBCs and IgM-only MBCs. "Non-classical" MBCs were also plotted against IgM and SSC to confirm that this population is mostly IgM+, and the IgM+IgD+ MBC population was selected and used in further analyses. These regions defined for the total (non-PS-specific) B cells were then used to define the PS-specific B cell subsets. The presented representative images are from the flow cytometric analysis of the PBMCs of one of the study's participants.*
